# Research on Improving the Durability of Bridge Pavement Using a High-Modulus Asphalt Mixture

**DOI:** 10.3390/ma14061449

**Published:** 2021-03-16

**Authors:** Wenfeng Wang, Shaochan Duan, Haoran Zhu

**Affiliations:** National Engineering Laboratory for Advanced Road Materials, JSTI Group, Nanjing 210017, China; nyscbwwf@163.com (W.W.); zhr75@jsti.com (H.Z.)

**Keywords:** bridge deck pavement, durability, high-modulus asphalt mixture, mechanical analysis, pavement performance

## Abstract

In order to improve the durability of the asphalt pavement on a cement concrete bridge, this study investigated the effect of the modulus of the asphalt mixture at the bottom layer on the mechanical response of bridge pavement, along with a type of emerging bridge pavement structure. In addition, the design method and pavement performance of a high-modulus asphalt mixture were investigated using laboratory and field tests, and the life expectancy of the deck pavement structure was predicted based on the rutting deformation. The results showed that the application of a high-modulus asphalt mixture as the bottom asphalt layer decreased the stress level of the pavement structure. The new high-modulus asphalt mixture displayed excellent comprehensive performance, i.e., the dynamic stability reached 9632 times/mm and the fatigue life reached 1.65 million cycles. Based on the rutting depth prediction, using high-modulus mixtures for the bridge pavement prolonged the service life from the original 5 years to 10 years, which significantly enhanced the durability of the pavement structure. These research results could be of potential interest for practical applications in the construction industry.

## 1. Introduction

During the 21st century, bridge construction has made considerable progress in China. By the end of 2018, the total number of national highway bridges had reached 851.5 thousand [1], among which, the cement concrete bridge is the most commonly used. After more than ten years of scientific research, a system of pavement materials with stone mastic asphalt (SMA), epoxy asphalt concrete, and waterproof adhesive layer materials with thermal sprayed modified asphalt, rubberized asphalt, and epoxy-based binder has formed [2,3,4,5]. However, in recent years, due to the increase in traffic volumes, there still exist many pavement diseases, for example, rutting, slippage, and potholes [6]. There is still a lot of improvement potential for the design of bridge pavement structures, pavement materials, and construction technology.

The existing research results showed that the high-modulus asphalt mixture had a high modulus, high rutting resistance, and low sensitivity to low-temperature cracking and fatigue cracking [7,8]. The above advantages could reduce the plastic deformation of the pavement structure, especially the rutting resistance at high temperatures when used in the middle and bottom pavement layers, and improve the pavement fatigue performance to extend its service life [9]. Based on the rerouting project of national highway G312 in the Zhenjiang urban area, this study systematically investigated the high-modulus asphalt mixture used in bridge pavement using mechanical analysis, laboratory and field tests, and a fatigue life prediction. The objective of this research was to improve the deformation resistance and antifatigue performance and durability of the bridge deck pavement structure, and finally, prolong the service life.

## 2. Raw Materials and Equipment

Hard asphalt is the key to improving the durability of a high-modulus asphalt mixture. Based on these characteristics, a petroleum asphalt #70 was produced by Petro China (Ningbo, China) and a hard and granular modifier (Verglimit V-260) which was from Switzeand Verglimit anti-icing road surface Co., Ltd. were chosen such that the mass ratio of the former to the latter was 7:3. Furthermore, the specimen consists of limestone with different particle sizes and mineral powders. The testing equipment to evaluate the road performance of the asphalt mixture mainly included a dynamic fatigue tester (YZM-T) which be collected from Tianjin Changji Test Instrument Technology Co., Ltd. in Tianjin, China, an automatic rutting tester (SYD-0719C-2) which be supplied by Tian Ce Technology Co., Ltd. in Nanjing, China, and a freeze-thaw splitting tester (TD729-2) purchased from Cangzhou Taiding Hengye Test Instrument Co., Ltd., China.

## 3. Pavement Structure Scheme and the Mechanical Analysis of Cement Concrete Bridge Deck

### 3.1. Bridge Pavement Structure Scheme

In China, the current cement concrete bridge deck asphalt pavements usually utilize double-layer pavement, which coincides with the intermediate and surface pavement layer of the connected roads [10]. The surface layer was 4 cm of modified asphalt SMA-13-M, and the lower layer was 6 cm of unmodified asphalt AC-20-U or modified asphalt AC-20-M. This paper presents a novel pavement structure scheme in which a high-modulus asphalt mixture (EME, for short; see below for the asphalt mix design) was used for the lower layer. The rutting test was used to evaluate the high-temperature stability of the durable and safe bridge deck pavement structure. The dynamic stability of the specimens with different structures is shown in Table 1.

ABAQUS was used to calculate the effect of using the high-modulus mixtures on the stress response of the pavement structure [11]. When using the finite element method to divide the mesh, in order to save time and improve the accuracy of the calculation, the C3D8R type was used in a box girder, a hollow slab girder, and concrete pavement. Through segmentation, adjusting the pattern of the mesh control to “structured” ensured the stability of the mesh division.

### 3.2. Development of Bridge Deck Pavement Model

**Figure 1:**
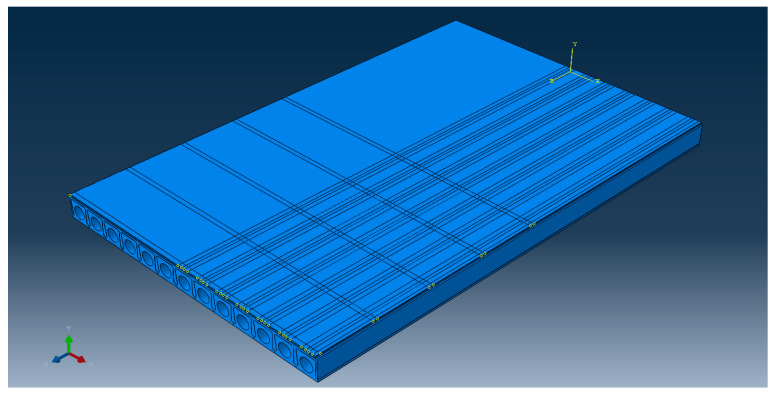
Structural model of the cement concrete bridge pavement.

According to the actual bridge structure used in the rerouting project of national highway G312 in the Zhenjiang urban area, this study selected the hollow slab girder as a representative bridge structure. The span, width, and other major size parameters of the bridge are shown in Table 2. For design temperatures from −80 to 200 °C, the 3D model of the hollow slab structure and the asphalt pavement layer was drawn according to the actual size using the finite element analysis software ABAQUS, as shown in Figure 1. In the ABAQUS modeling process, the transient elastic parameter “**Moduli time scale**” was selected as “**Instantaneous**”, and the viscoelastic parameter was defined as the “**Prony series**” for the time.

### 3.3. Material and Load Parameter Selection

Asphalt pavement materials are considered uniform, isotropic viscoelastic mixtures [12]. A generalized Maxwell model was selected to represent the viscoelastic mechanical behavior of asphalt mixtures [13]. The viscoelastic parameters of SMA-13-M, AC-20-U, AC-20-M, and EME were determined on the basis of dynamic modulus tests (ASTM D3497) [14]. The Poisson’s ratio of the asphalt mixture was taken as 0.25. A cement bridge deck and a waterproof bonding layer were used in the linear elastic model. The Poisson’s ratio of the concrete bridge deck plate was 0.35 and the elastic modulus of the concrete was 32.5 GPa. The thickness of the waterproof bonding layer was 0.2 cm and the elastic modulus was 150 MPa. The physical properties of the asphalt mixture are shown in Table 3.

### 3.4. Analysis of the Most Unfavorable Load Position

Due to the various positive and negative bending moment factors, the load position has an important influence on the mechanical response of a concrete deck pavement system. Selecting the most unfavorable load position is the key to accurately calculating the maximum load stress in the pavement system [15].

Transverse load position: The rerouting project adopted a two-way, six-lane layout, where the single width of lane was set to 12.5 m. In order to research the influence of the lateral arrangement of a vehicle on the pavement layer stress, by considering the bridge symmetry and the actual situation of a vehicle moving on the bridge, the analysis started from the middle of the slab, moving 1 m as the analysis step length toward the margin, which resulted in seven positions.

Longitudinal loading position: By considering the symmetry of the longitudinal load along the bridge, the loading positions were selected at the bridge support, *L*/8, *L*/4, 3*L*/8, and *L*/2, where *L* was the length of the bridge.

By considering the pavement damage mechanism, the selected mechanical control indexes were the maximum shear stress and the horizontal tensile stress in the pavement, the vertical tensile stress between the asphalt concrete and the cement concrete layer, and the maximum horizontal shear stress between the asphalt concrete and the cement concrete layers.

#### 3.4.1. Most Unfavorable Transverse Position

**Figure 2:**
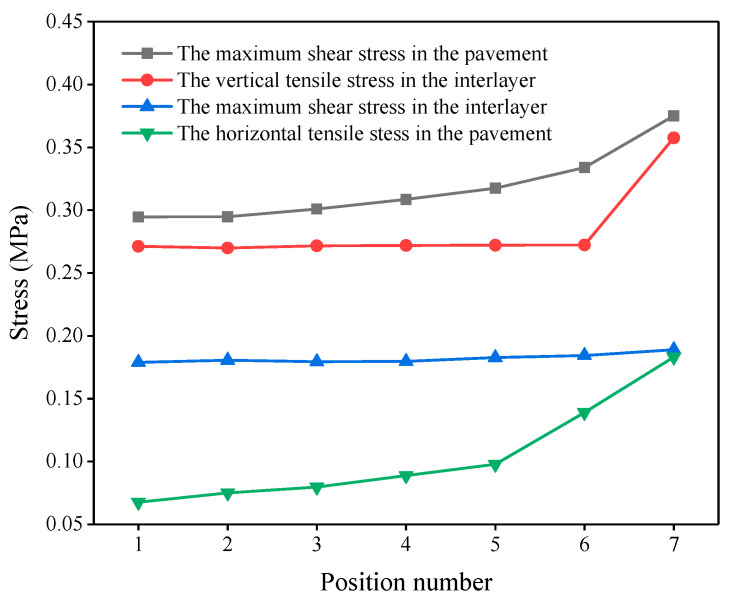
Lateral stresses in the pavement at different loading positions.

The mechanical analysis results of the deck pavement with different lateral load positions are shown in Figure 2. It can be seen that the maximum shear stress and the horizontal tensile stress in the pavement gradually increased with the loading position shifting from the bridge’s center (position 1) to the bridge’s edge (position 7). Furthermore, the maximum value appeared at the edge of the bridge. The vertical tensile stress at the bridge’s edge (position 7) increased significantly, while the other stress indexes did not change like this. Therefore, the bridge’s edge (position 7) was selected as the most unfavorable position in the transverse direction.

#### 3.4.2. Most Unfavorable Longitudinal Position

**Figure 3:**
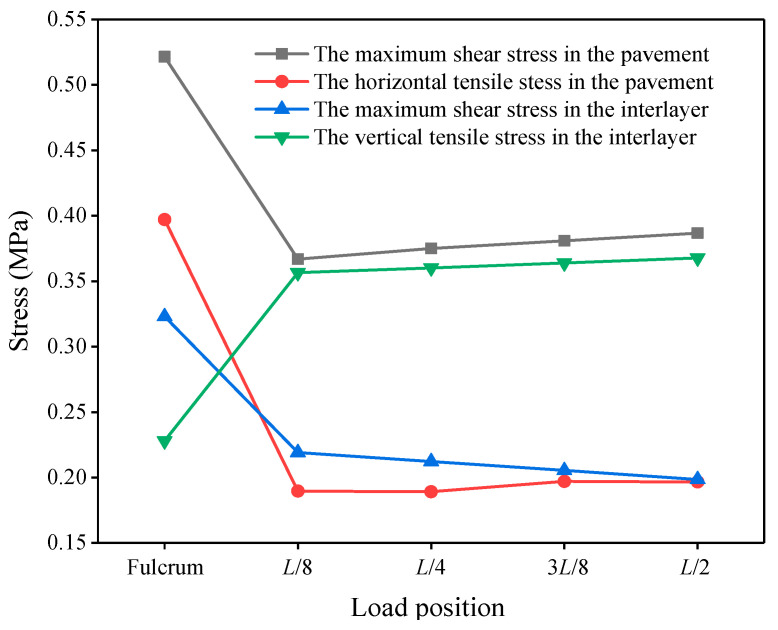
Longitudinal stresses in the pavement at different loading positions.

The mechanical analysis results of the deck pavement with different longitudinal load positions are shown in Figure 3. The maximum shear stress and the horizontal tensile stress in the pavement, as well as the maximum horizontal shear stress in the interlayer, at the fulcrum were obviously higher than that at the other positions. It can be seen from the three indicators above that the most unfavorable longitudinal position was at the bridge support, mainly due to the strong bearing support at the bridge’s end, which was prone to generating a greater negative bending moment, which produced greater stress in the layer. Therefore, the support position was selected as the most unfavorable position in the longitudinal direction.

### 3.5. Mechanical Response Analysis of the Deck Pavement with Different Paving Materials

The dynamic modulus and phase angle of several asphalt mixtures at 20 °C are shown in Table 4. The order of the dynamic moduli from small to large was AC-20-U < AC-20-M < EME.

**Figure 4:**
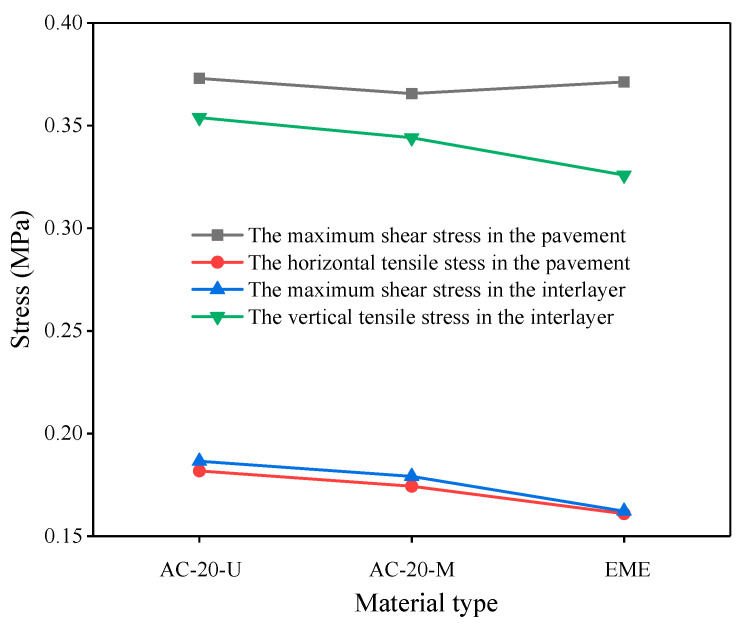
The stress responses in the bottom layer of various pavement structures.

On the basis of the analysis of the most unfavorable loads on the hollow slab in the longitudinal and transverse directions, the AC-20-U, AC-20-M, and EME were used in the lower pavement layer. The results of mechanical responses were analyzed and are shown in Figure 4.

It can be seen from Figure 4 that with the increase of the asphalt mixture modulus, the maximum shear stress in the pavement was almost unchanged. The transverse tensile stress in the pavement, the horizontal shear stress at the interlayer, and the vertical tensile stress at the interlayer showed a downward trend. This was attributed to the fact that with the modulus increase of the lower layer pavement, the stiffness was also increasing, which alleviated the effect of the bridge deck’s deformation on the pavement structure, which decreased the lateral tensile stress within the pavement structure [16,17]. On the other hand, since the difference between the modules of the pavement and the concrete bridge structure was reduced, the maximum horizontal shear stress and vertical tensile stress between the layers were reduced [18]. The high-modulus asphalt pavement mixture made the whole structure of the deck pavement system more coordinated, hence achieving the purpose of improving the pavement stress state.

## 4. Design and Performance Study of the High-Modulus Asphalt Mixture

### 4.1. Design of the High-Modulus Asphalt Mixture

The concept of high-modulus asphalt mixtures was first proposed in France [19,20]. The mixture was designed based on both performance indexes and mechanical properties, using low-grade asphalt or hard asphalt, a high oil–stone ratio, and particular gradation to produce a smaller interspace. The gyratory compaction method was adopted. Antimoisture damage tests and wheel tracking tests were used to represent the road performance [21,22]. In addition, dynamic modulus tests and fatigue resistance tests were used to measure the mechanical properties [23].

The asphalt content was selected based on an indicator named the richness modulus in the French EME design method, which is different from the design methods according to volumetric indicators used in the France and China [24,25].

#### 4.1.1. Selection of Raw Materials

Low-grade asphalt or hard asphalt is the key material that is required to achieve a high-modulus asphalt mixture [26]. Based on existing experience, unmodified asphalt and hard particles can be blended to produce low-grade asphalt. Hard particles are high-viscosity asphalt products that are made from heavy crude oil as the raw material through special processing that involves high-vacuum distillation, removing the solvent, oxidation, and a blending process. The weight ratio of 7:3 was chosen as the ratio of unmodified asphalt to hard particles. The test results are shown in Table 5, which met the requirements of EN 13924 for hard asphalt of grades 15~25.

#### 4.1.2. Gradation and Asphalt Content

**Figure 5:**
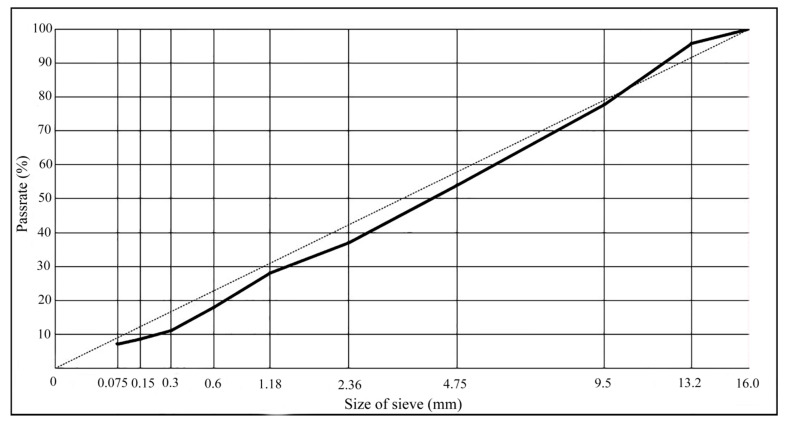
Aggregate gradation curve.

According to the results of the mineral aggregate (basalt) screening, a gradation was synthesized according to the proportion of 1 # (5–15 mm): 2 # (3–5 mm): 3 # (0–3 mm): mineral powder = 42.0:15.0:41.0:2.0. The synthetic gradation curve is shown in Figure 5.

Using the above gradation, the abundance coefficient *K* was 3.59 when the asphalt/stone ratio was 5.7%, which met the requirement of the abundance coefficient *K* of 3.4.

The French gyratory compaction test was carried out in accordance with the French specification (NF P 98-252) using the gradation and asphalt–stone ratio described above [27,28]. The results of the gyratory compaction tests are compiled in Table 6. According to the result of the rotary compaction test, the porosity (2.8%) of the high-modulus asphalt mixture met the design requirements.

### 4.2. Pavement Performance Evaluation

#### 4.2.1. Moisture Susceptibility

According to the freeze–thaw splitting test JTG E20 T 0729-2000 [29], the split strength ratio of the mixture specimens before and after the moisture damage was used to evaluate the moisture susceptibility of the high-modulus asphalt mixture. It can be seen from Table 7 that the freeze–thaw splitting strength ratio (TSR) of the high-modulus asphalt mixture was 86.2%, which satisfied the specification technical requirements. Meanwhile, the splitting strength of the high-modulus asphalt mixture was 2 to 3 times higher than the ordinary asphalt mixture, the strength of which was 0.8~1.0 kN.

#### 4.2.2. High-Temperature Stability

##### Chinese Rutting Test

The high-temperature stability of the high-modulus asphalt mixture was tested using the Chinese wheel tracking test method JTG E20 T 0719-2011 [29], which was compared with the traditional modified asphalt mixture AC-20. The test conditions were 60 ± 1 °C and 0.7 ± 0.05 MPa.

It can be seen from Table 8 that the dynamic stability of the high-modulus asphalt mixture was 9632 times/mm, which was 30% higher than that of the modified asphalt mixture AC-20. The durability of the high-modulus asphalt mixture was demonstrated by its excellent high-temperature resistance to rutting [30].

##### French Rutting Test

The high-temperature stability of the high-modulus asphalt mixture was determined using the French wheel tracking test NF P 98-252 (corresponding to EN 12697-22) [31]. The results of the test are shown in Table 9. It can be seen that the rutting ratio gradually increased with the increase of the number of load cycles. When the number of load cycles was 30,000, the rutting rate still met the requirements, which shows that the high-modulus asphalt mixture displayed excellent high-temperature stability.

#### 4.2.3. Fatigue Performance

Two-point trapezoidal beam tests were carried out according to the French specification NF P 98-261-1 (EN 12697-24 Annex A) [32] to measure the fatigue behavior of the high-modulus asphalt mixture. It was tested under the following conditions: 10 °C, 25 Hz, and 130 με.

It can be seen from Table 10 that the fatigue life of the durable high-modulus asphalt mixture reached 1.65 million cycles at 130 με, far more than the technical requirements of 1 million cycles. Therefore, the high-modulus asphalt mixture displayed excellent fatigue properties.

#### 4.2.4. Mechanical Properties

Two-point trapezoidal beam modulus tests were conducted according to the French specification NF P 98-260-2 (corresponding to EN 12697-26 Annex A) to evaluate the mechanical properties of the high-modulus asphalt mixture [33]. The test temperature and frequency were 15 °C and 10 Hz, respectively. It can be seen from Table 11 that the complex modulus of the high-modulus asphalt mixture reached 19,620 MPa, which satisfied the technical requirement of more than 14,000 MPa.

In summary, in order to improve the durability and rutting resistance of the pavement structure under a load, a high-modulus asphalt mixture with high strength, a high modulus, and excellent high-temperature stability and fatigue resistance can be used for the lower layer of the bridge pavement.

## 5. Validation of the Practical Project

In the rerouting project of national highway G312 in Zhenjiang urban area, in order to further improve the construction quality and promote the application of new technologies and new materials, a trial pavement structure with high-modulus mixtures was constructed in 2016 at the Shengyuan Road interchange to enhance the durability of the bridge pavement.

In order to demonstrate the actual construction effect, the water permeability test and compaction degree test were carried out on the test section.

Table 12 reveals that the qualified rate found using the water permeability test was 100%. Moreover, both the theoretical compaction degree and the rotational compaction degree of the core samples met the technical requirements.

**Figure 6:**
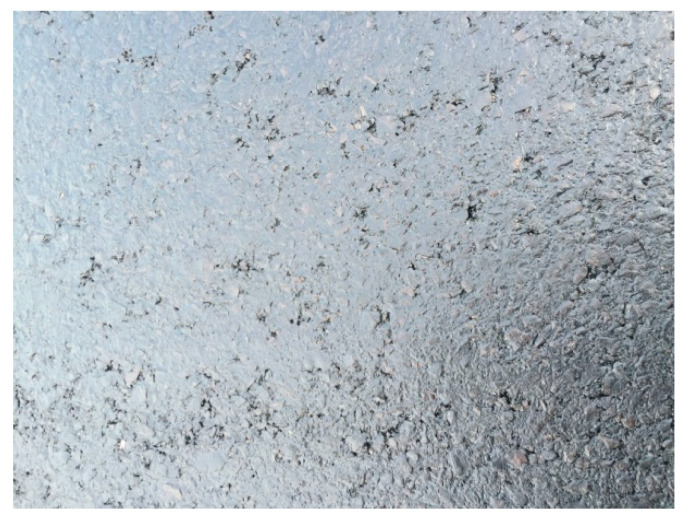
Effect of paving using the high-modulus asphalt mixture.

As shown in Figure 6, the high-modulus asphalt mixture pavement had a smooth surface, and the compaction, water permeability, and other indicators met the requirements. The practical engineering application proved that the construction effect of the high-modulus mixture pavement was successful.

## 6. Life Prediction of the Deck Pavement Based on the Rutting Deformation

In order to verify the durability of the high-modulus asphalt mixture used in the lower layer of bridge pavement, the rutting deformation was taken as the control index and the calculation formula for the rutting depth in the Specifications for Design of Highway Asphalt Pavement (JTG D50-2017) was used in this paper [34]. When the lower layer was made of durable high-modulus EME-14 and AC-20-M, the permanent deformation of the asphalt pavement structure reached the design-allowable value of 10 mm

According to the layering method, each asphalt pavement structure was divided into five layers according to the following layer thicknesses: 40 mm thick rubber asphalt SMA-13 upper layer was divided into 20 + 20 mm and a 60 mm thick durable high-modulus EME-14 or 60 mm AC-20-M bottom layer was divided into 20 + 20 + 20 mm.

The vertical compressive stresses at the top of each layer under the designed load were calculated. The results are provided in Table 13.

The latest data found that the average daily equivalent axle load and annual growth rate of vehicle flow of the G312 highway were 6086 times/day and 6.19%. Thus, the cumulative number of axle loads for different design years was calculated [35]. The permanent deformation of the pavement structure corresponding to different design years is shown in Table 14.

According to the results, for the traditional pavement structure using AC-20-M, the rutting depth will reach 10.34 mm after 5 years. However, for the durable pavement structure using EME-14, the rutting depth will reach 10.67 mm after 10 years. Therefore, the service lives are expected to be 5 and 10 years, respectively, when a rutting depth of 10 mm is taken as the requirement.

The high-modulus asphalt mixture technology can improve the durability of the deck pavement, effectively reduce the probability of rutting, and consequently, it will extend the maintenance interval and reduce the traffic delays due to maintenance work during the operation period.

## 7. Conclusions

In this study, through mechanical analysis, laboratory and field tests, and life prediction, a high-modulus asphalt mixture that was designed and used as the bottom layer of a cement concrete bridge pavement system was investigated; the main conclusions are as follows:

(1) When the high-modulus mixture was used for the lower layer of the bridge deck pavement, the difference in modulus between the pavement layer and the concrete bridge was reduced. As a result, the whole structure of the deck pavement system was more coordinated, achieving the purpose of improving the pavement stress state.

(2) The road performance test results showed that with its high strength and modulus and excellent high-temperature stability and fatigue resistance, the high-modulus asphalt mixture can be used for the lower layer of deck pavement to improve the ability to resist rutting, reducing the permanent deformation under loading.

(3) The results of the practical engineering application suggested that the new high-modulus asphalt mixture had excellent comprehensive performance, i.e., the dynamic stability reached 9632 times/mm and the fatigue life reached 1.65 million cycles. However, the long-term performance needs to be further tracked and evaluated.

(4) The life prediction based on the rutting deformation revealed that when the deck pavement structures with EME and AC-20-M were compared, the times to reach 10 mm rutting depth are expected to be 10 and 5 years, respectively. The application of the high-modulus asphalt mixture can significantly improve the durability of bridge pavement.

## Figures and Tables

**Table 1:** Pavement structure scheme.

Structure	4 cm SMA-13-M 6 cm AC-20-U	4 cm SMA-13-M 6 cm AC-20-M	4 cm SMA-13-M 6 cm EME
Dynamic stability (time/mm)	5478	5705	6562

EME: the high-modulus asphalt mixture.

**Table 2:** Main size parameters of the hollow slab.

Items	Parameters
Bridge span arrangement	20 m
Bridge type and structural system	Prestressed concrete hollow slab girder
Thickness of the concrete bridge deck	10 cm
Hollow plate height	90 cm
Diameter of the hole in the plate	59 cm
Space between the holes	99 cm
Width (one deck)	12.5 m

**Table 3:** Physical properties of the asphalt mixture.

Asphalts	Modulus	0.01 Hz	0.1 Hz	1 Hz	10 Hz
SMA-13-M	*E* (kPa)	7110.4	6399.5	3537.5	4854.8
	*G* (kPa)	2844.1	2559.8	1415	1941.9
	*K* (kPa)	4740.2	4266.3	2358.3	3236.5
AC-20-U	*E* (kPa)	6142.2	5009.8	3045	3241.4
	*G* (kPa)	2456.8	2003.9	1218	1296.5
	*K* (kPa)	4094.8	3339.8	2030	2160.9
AC-20-M	*E* (kPa)	825.8	6287.8	3594.3	3882.4
	*G* (kPa)	330.32	2515.1	1437.7	1552.9
	*K* (kPa)	550.5	4191.8	2396.2	2588.2
EME	*E* (kPa)	0	7521.9	4677.2	7880.4
	*G* (kPa)	0	3008.7	1870.8	3152.1
	*K* (kPa)	0	5014.6	3118.1	5253.6

*E*: Elastic modulus, *G*: Shear modulus, *K*: Bulk modulus.

**Table 4:** Dynamic modulus and phase angle test results of the various asphalt mixtures.

Type	Parameters	Frequency (Hz)					
		0.1	0.5	1.0	5.0	10	25
AC-20-U	Dynamic modulus (MPa)	2682	4162	5087	8107	9258	11,421
	Phase angle (°)	30.33	28.75	25.56	20.34	18.43	14.21
AC-20-M	Dynamic modulus (MPa)	2922	5142	6387	9547	11,214	13,550
	Phase angle (°)	33.90	30.12	28.25	21.40	18.92	15.68
EME	Dynamic modulus (MPa)	6031	9645	11,344.5	15,433.5	17,332.5	19,863
	Phase angle (°)	33.90	30.12	28.25	21.40	18.92	15.68

**Table 5:** Hard asphalt key indicators.

Key Indexes	Test Results	Technical Requirements
Penetration 25 °C, 100 g, 5 s (0.1 mm)	15	15~25
Softening point (°C)	66.0	≥60
60 °C dynamic viscosity (Pa·s)	4580.3	≥3500
Rolling thin film oven test (RTFOT)		
Mass change (%)	−0.05	≤±0.5
Residual penetration ratio (25 °C)	81.5	≥55

**Table 6:** Results of the gyratory compaction test (100 cycles).

No.	Height (mm)	Mass in Air (g)	Mass in Water (g)	Surface Dry Mass (g)	Bulk Specific Gravity	* Porosity with French Standard (%)
1	114.1	4838.4	2855.5	4841.3	2.436	2.8
2	113.9	4826.9	2850.9	4830.9	2.438	

* Porosity calculation equation according to the French standard: *VV* = (1 − *h*_min_/*h_i_*) × 100, where *VV*—porosity (%), *h*_min_—the minimum specimen height at which the porosity was zero (mm), and *h_i_*—specimen height after the gyratory compaction of *i* cycles (mm).

**Table 7:** Results of freeze–thaw splitting test.

Asphalt Mixture	Without the Freeze–Thaw Cycle		After the Freeze–Thaw Cycle		TSR (%)	Technical Requirements (%)
	Porosity (%)	Splitting Strength (MPa)	Porosity (%)	Splitting Strength (MPa)		
EME-14	4.0	2.2860	3.7	1.8802	86.2	≥75
	3.9	2.1282	3.8	1.8930		
	3.8	2.3386	4.2	1.9841		
	4.1	2.1441	4.0	1.9140		
Average	4.0	2.2242	3.9	1.9178		
AC-20-M	5.3	1.0202	5.4	0.8515	86.7	
	5.1	0.9925	5.2	0.8766		
	5.3	1.0363	5.5	0.8859		
	5.4	1.0055	5.1	0.9010		
Average	5.3	1.0136	5.3	0.8788		

TSR: freeze–thaw splitting strength ratio.

**Table 8:** Results of the Chinese wheel tracking test.

Asphalt Mixture	Porosity (%)	Dynamic Stability (times/mm)					Coefficient of Variation (%)	
		1	2	3	Average	Requirements	Result	Requirements
EME-14	1.9	9265	10,500	9130	9632	≥2800	7.8	≤20
Modified asphalt AC-20	4.6	7590	7412	6923	7308		4.7	

**Table 9:** Results of the French rutting test.

No.	Rutting Ratio at Different Loading Cycles (%)						Requirements (%)
	100	300	1000	3000	10,000	30,000	
1	2.19	2.43	3.09	3.35	4.00	4.44	30,000 times ≤7.5
2	1.89	2.53	3.33	4.03	4.62	5.02	
Average	2.04	2.48	3.21	3.69	4.31	4.73	

**Table 10:** Results of the fatigue performance test.

Temperature (°C)	No.	Porosity (%)	Results (Cycles)	Requirements (Cycles)
10	1	2.6	1,606,609	≥10^6^
	2	2.9	1,136,415	
	3	2.7	1,935,684	
	4	3.0	1,935,684	
Average		/	1,653,598	

**Table 11:** Results of the dynamic modulus test.

Temperature (°C)	No.	Porosity (%)	Complex Modulus (MPa)	Requirements (MPa)
15	1	2.6	19,131	≥14,000
	2	2.9	19,594	
	3	2.7	19,498	
	4	3.0	20,259	
Average		/	19,620	

**Table 12:** Results of high-modulus asphalt pavement.

No.	Water Permeability Coefficient (mL/min)	Theoretical Compaction Degree (%)	Gyratory Compaction Degree (%)
Point 1	0	98.6	99.8
Point 2	6	98.1	99.3
Point 3	0	98.9	100.1
Point 4	0	98.8	100.0
Point 5	10	98.7	99.9
Requirements	≤50	96~99	≥98

**Table 13:** Calculated compressive stress at the top of different layers.

Pavement Structure Type	Point 1	Point 2	Point 3	Point 4	Point 5
EME-14 bottom layer	0.707	0.627	0.547	0.507	0.467
AC-20-M bottom layer	0.707	0.624	0.549	0.512	0.484

**Table 14:** Permanent deformation of different pavement structures

Pavement Structure Type	Designed Life (Years)	Accumulated Equivalent Axle Loadings (Million)	Permanent Deformation of Each Layer (mm)					Total Rutting Deformation (mm)
			*R*_1_	*R*_2_	*R*_3_	*R*_4_	*R*_5_	
EME-14 bottom layer	10	2.954 × 10^5^	1.02	2.67	3.06	2.35	1.57	10.67
AC-20-M bottom layer	5	1.257 × 10^5^	0.68	1.76	3.41	2.64	1.85	10.34

*R*: Repetition.

## Data Availability

Data is contained within the article.
